# Income and campus application disparities among European and non-European heritage Hispanic undergraduate applicants

**DOI:** 10.1093/pnasnexus/pgae337

**Published:** 2024-08-21

**Authors:** AJ Alvero, Sonia Giebel, Francis A Pearman

**Affiliations:** Center for Data Science for Enterprise and Society, Cornell University, Ithaca, NY 14850, USA; Global Sociology, Social Science Center Berlin (WZB), 10785 Berlin, Germany; Graduate School of Education, Stanford University, Stanford, CA 94305, USA

**Keywords:** Hispanic, college admissions, disaggregation, ethnicity

## Abstract

Leveraging every undergraduate application submitted by self-identified Hispanic applicants to the University of California system in the 2016 and 2017 application cycles, we show that a significant number of applicants claim Hispanic identity by virtue of European heritage. We subsequently demonstrate that Hispanic-identifying students of European descent are significantly more affluent and more likely to apply to selective University of California campuses than their non-European Hispanic peers. We comment on the practical implications of these disparities, as well as their relevance for studies of inequality in the social sciences and education.

## Introduction

In the United States, the term “Hispanic” collapses many different ethnicities from Latin American and the broader Hispanophone world into one panethnic category (1). In the aggregate, Hispanic peoples face persistent forms of social inequality in the United States, including higher rates of poverty, lower rates of educational attainment, and racist associations (2). Many social scientists accordingly use “Hispanic” as a single ethnoracial marker in studies of inequality, oftentimes comparing Hispanic and Black populations in the United States (3) because they perceive both groups to face similar forms of inequality. Yet, these inequalities do not affect members of the Hispanic category uniformly, but are reflective of broader inequalities disproportionately affecting Latin American and other “Global South” countries (4). Some institutions have adjusted their policies in light of these dynamics. For example, the former Ford Foundation Fellowship explicitly noted their preference for Hispanic applicants of Mexican and Puerto Rican heritage, given the degree of underrepresentation in the US academy relative to their US populations (5). However, the Ford Foundation is largely an anomaly.

Instead, the ambiguity of the category, including those of varying immigrant backgrounds, phenotypes, and linguistic heritage, allows for broad claim to the identity (1). The stakes of who claims the Hispanic identity increase when material resources are implicated. For example, online forums about college admissions strategies encourage a capacious definition of who “counts” as Hispanic and generate ample justification (6). Subsequently, individuals with varying social backgrounds and understandings of “Hispanic” may all claim the identity, increasing variation within the category. Disaggregation sharpens social inquiry by more precisely identifying patterns by specific subgroups (7, 8), as demonstrated by scholars of health and demography (9). Administrative data in key social institutions, such as those held by selective college admissions offices, provide much needed further insight into heterogeneity within US Hispanic populations.

In this article, we provide an illustration of the importance of showing variation within the Hispanic category. We reveal that a significant number of University of California (UC) applicants claim Hispanic identity by virtue of European ancestry, and that these students have higher incomes and are more likely to apply to the more selective (and well-resourced) UC campuses. We furthermore show application rates by income for both European heritage and non-European heritage applicants that largely correspond to campus selectivity. We draw on every Hispanic-identifying application to the UC system in the 2016 (n=44,434) and 2017 (n=48,702) application cycles to illustrate this phenomenon. Our findings speak to how ethnoracial categorizations impact the intra-enthnoracial diversity of student populations in higher education, with particular relevance for the governance of Hispanic Serving Institutions (HSIs).

## Results

### European heritage a plurality among “other” respondents

Of the Hispanic applicants using the “Other” category (n=11,066 or 12% of all Hispanic UC applicants), 40% reported some kind of European heritage (n=4,466). We refer to these students hereafter as European Heritage (EH) students. This proportion was larger than the biggest subgroup of applicants not mentioning European heritage: 35% of “Other” respondents referenced Central American heritage (n=3,959). 82,070 students did not reference Europe. We refer to these students as non-European Heritage (NEH) students. EH applicants referred to countries (“Spain,” “Portugal,” “France,” and “Italy”) and provided genealogical justifications (e.g. “Born in Florida to Spaniards,” “38% Iberian Peninsula”^a^). Given that the UC does not consider race or ethnicity in its admissions protocols, and that the absence of affirmative action lowers the likelihood of minority group identification (10), the self-identifications offered are likely earnest representations of Hispanic identity.

### Income disparities between EH and NEH applicants

**Fig. 1.**
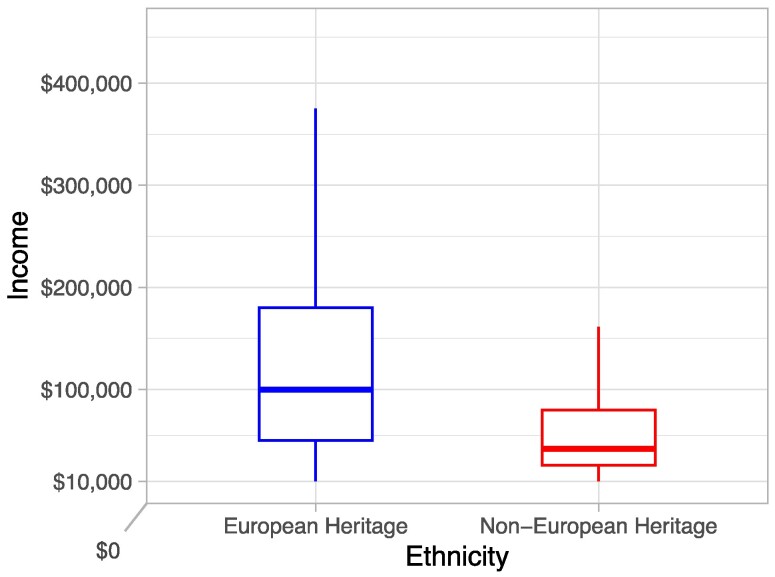
Boxplots comparing income for EH and NEH Hispanic applicants.

EH applicants were far more affluent than NEH applicants. Figure 1 visualizes income differences by group. The median household income for EH-Hispanic applicants ($100,000) was more than double the median income of all other Hispanic-identifying applicants ($42,000) in the sample. The mean income for NEH applicants in the sample was $70,591; the mean income for EH applicants was $142,453.

### Differences in campus preference between EH and NEH applicants, by income level

**Fig. 2.**
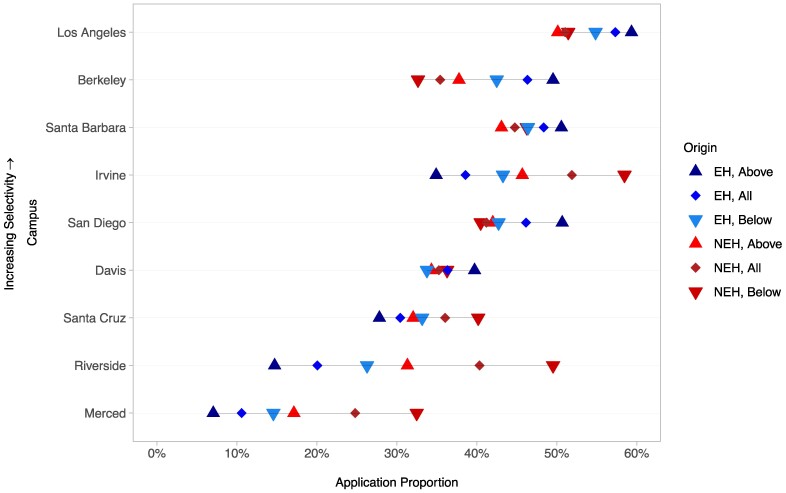
Proportion of EH and NEH applicants applying to each UC campus, by income level (above or below median, all), ordered by campus selectivity.

Figure 2 plots the campus application rates of EH applicants (blue shapes) and NEH applicants (red shapes), broken down by income level. EH Hispanic applicants were more likely to apply to the most selective campuses (Los Angeles, Berkeley, and Santa Barbara), while NEH Hispanic applicants were more likely to apply to the least (Santa Cruz, Riverside, and Merced). Of the remaining campuses, NEH applicants were more likely to apply to Irvine, and EH applicants were more likely to apply to San Diego and Davis. Said differently, it is at Davis’ selectivity that EH applicants apply in greater proportions than NEH applicants, with the exception of Irvine.

Accounting for income levels largely reinforces these disparities: seven out of nine UC campuses exhibit classed application rates corresponding to campus selectivity. At UCLA, Berkeley, UCSB, and UCSD, application rates increase with income, meaning high-income EH applicants apply at the highest rates and low-income NEH applicants at the lowest; at UCSC, Riverside, and Merced, application rates *decrease* with income, meaning lower-income NEH students apply at the highest rates, and higher-income EH applicants at the lowest. Berkeley and Riverside are illustrative examples of this phenomenon: nearly 50% of EH applicants with the highest incomes applied to Berkeley; in contrast, 33% of NEH applicants with the lowest incomes applied to the State flagship. At the other end of the selectivity spectrum, 50% of the lowest-income NEH applicants applied to Riverside; just 15% of the highest income EH applicants did the same. Looking across all campuses, EH students generally apply at greater rates to more selective campuses, while NEH students apply disproportionately to less selective campuses, with income levels amplifying these inter-ethnic trends.

### Discussion

We have illustrated the importance of disaggregating Hispanic data by showing that European heritage Hispanic students are significantly wealthier and more likely to apply to selective UC campuses than their non-European heritage peers. These findings complicate efforts to improve higher education access for students from “underrepresented backgrounds,” which often include *all* students who identify as Hispanic. The prominence of European references in the data also complicates notions of race in the United States, reflecting differing degrees of “Europeanness” vs. “non-Europeanness” (11). Our results should encourage researchers already attentive to heterogeneity within the category, in fields ranging from health (9), computational social science (12), and economic sociology (13), to consider European heritage as an additional dimension of variation.

Our work naturally speaks to universities seeking to become HSIs. HSIs are degree-granting colleges and universities that enroll at least 25% Hispanic-identifying students; they receive federal financial support upon achieving this designation (14, 15). All UC campuses have publicly declared their intent to become HSIs.^b^ We recognize that EH applicants constitute a small proportion (5%) of our data. However, EH students may still play an important role in achieving HSI designation. Berkeley has declared its intent to become an HSI by 2027. Its Hispanic enrollment is currently 6,795 (20.5% of its 33,078 undergraduates), or 1,475 students short of the 8,270 needed to reach the 25% threshold. In Fall 2023, 211 students identifying as “Other Spanish American/Latino” enrolled at Berkeley.^c^ Should 40% of those students have noted European heritage, as was the case in our data, that would yield 85 EH students. While 85 is small relative to Berkeley’s total enrollment, it is significant relative to its Hispanic enrollment goals: To become an HSI by 2027, Berkeley will need to enroll 370 Hispanic-identifying students *over* its current enrollment trends per year, increasing the meaningfulness of those hypothetical 85 EH students. Additionally, UC enrollment data shows differences in the Hispanic sub-populations across the UC: in the fall enrollment terms corresponding to our data, about 14% of Hispanic Berkeley matriculants identified as “Other Spanish American/Latino,” whereas less than 8% of Merced matriculants identified as such. Though our data cannot speak directly to admissions or matriculation patterns, these enrollment figures suggest the application disparities we surface are likely to remain after the admissions process.

Enrollment aside, application rates reflect where students envision themselves in the future. Prior academic achievement certainly contributes to where students apply. We unfortunately do not have access to academic metrics but acknowledge that academics likely explain some of the patterning we see, especially in light of the strong correlation between academic achievement and income (16). Still, application rates are also reflective of where students see themselves belonging (14). The fact that selective campuses largely attract wealthier EH students and the less selective campuses—those already designated as HSIs—attract lower-income NEH students suggests that EH and NEH students view the UC campuses in distinct lights. They also seem to aspire to them in ways that echo existing classed and racialized hierarchies in the UC system (17).

Our intention is not to encourage boundary policing nor reject studies highlighting inequalities faced by Hispanic populations. Rather, we show how the construction of the category complicates social opportunity goals associated with it.^d^ “Hispanic” is not the only category to which this caveat theoretically applies. Consider a scenario where “Caribbean” were a panethnic category in the United States meant to describe people from former British colonies like Jamaica or The Bahamas. The term could similarly represent those who have faced particular hardships and to communicate ethnoracial diversity in organizations. In this scenario, people could claim “Caribbean” membership via British heritage, distorting the original intent of the category and the data. The current configuration of “Hispanic” is not much different, yet little comparable research has considered the degree to which European identities comprise the category. We urge our fellow social scientists to take up this call.

## Materials and methods

Our data comprise all UC applications submitted in the 2015–2016 and 2016–2017 academic years. We combine all data for analysis (n=93,106). The data include every Hispanic-identifying first-year applicant, excluding transfer applicants. Applicants were able to select Mexican, Cuban, Puerto Rican, or “Other.” We did not have access to any other ethnoracial information (e.g. “Black”), nor academic achievement (e.g. GPA or standardized test scores). In total, 11,066 applicants selected “Other” and provided open-ended responses (5,236 in 2015–2016 and 5,830 in 2016–2017). Responses were labeled as EH if they included explicit references to European countries or regions. We also included colonial identities rooted in the US, such as “Californio,” “New Mexican,” and “10th Generation Californian” given their direct colonial affiliation to Spain (18). Applicants who referenced Europe and another identity were labeled as “European identifying.” Given that little at this point is known about the relative standing of European heritage Hispanics of any kind or degree, we opted for this more inclusive definition of European heritage. Future studies could address the standing of multiracial Hispanics, especially in light of new Census guidelines (19). Applicants who did not report an income were excluded from any pertinent analyses. We follow past work and also exclude applicants who reported an income below $10,000 (16) (total excluded due to income below $10,000 and no reported income = 5,383; 145 identified as European).

## Data Availability

The data to replicate our findings are here.

## Notes

a The partial and genealogical identifications contrast with a Pew study showing that identifying as Hispanic decreases for subsequent generations: https://pewrsr.ch/2BphFwM

b
https://www.ucop.edu/hsi-initiative/index.html

c
https://www.universityofcalifornia.edu/about-us/information-center/disaggregated-data

d The revised Census guidelines include “Hispanic or Latino” as a response to a combined race/ethnicity question, but the definition of the category is unchanged: “Includes individuals of Mexican, Puerto Rican, Salvadoran, Cuban, Dominican, Guatemalan, and other Central or South American or Spanish culture or origin.” https://www.census.gov/newsroom/blogs/random-samplings/2024/04/updates-race-ethnicity-standards.html

## Preprint

Preprint version: https://osf.io/preprints/edarxiv/ske5m.
